# High-Throughput Screening Campaign Identified a Potential Small Molecule RXFP3/4 Agonist

**DOI:** 10.3390/molecules26247511

**Published:** 2021-12-11

**Authors:** Guangyao Lin, Yang Feng, Xiaoqing Cai, Caihong Zhou, Lijun Shao, Yan Chen, Linhai Chen, Qing Liu, Qingtong Zhou, Ross A.D. Bathgate, Dehua Yang, Ming-Wei Wang

**Affiliations:** 1 The National Center for Drug Screening, Shanghai Institute of Materia Medica, Chinese Academy of Sciences, Shanghai 201203, China; linguangyaomail@163.com (G.L.); fengyang1209@163.com (Y.F.); xqcai@simm.ac.cn (X.C.); z_rainbow@aliyun.com (C.Z.); lhchen@simm.ac.cn (L.C.); qliu@simm.ac.cn (Q.L.); 2 University of Chinese Academy of Sciences, Beijing 100049, China; shaolj@shanghaitech.edu.cn; 3 School of Life Science and Technology, ShanghaiTech University, Shanghai 201210, China; 4 Department of Pharmacology, School of Basic Medical Sciences, Fudan University, Shanghai 200032, China; 19111030075@fudan.edu.cn (Y.C.); zhouqt@fudan.edu.cn (Q.Z.); 5 Florey Institute of Neuroscience and Mental Health and Department of Biochemistry and Pharmacology, The University of Melbourne, Parkville, VIC 3052, Australia; bathgate@florey.edu.au; 6 The CAS Key Laboratory of Receptor Research, Shanghai Institute of Materia Medica, Chinese Academy of Sciences, Shanghai 201203, China; 7 Research Center for Deepsea Bioresources, Sanya 572025, Hainan, China

**Keywords:** RXFP3, RXFP4, high-throughput screening, agonist, WNN0109-C011

## Abstract

Relaxin/insulin-like family peptide receptor 3 (RXFP3) belongs to class A G protein-coupled receptor family. RXFP3 and its endogenous ligand relaxin-3 are mainly expressed in the brain with important roles in the regulation of appetite, energy metabolism, endocrine homeostasis and emotional processing. It is therefore implicated as a potential target for treatment of various central nervous system diseases. Since selective agonists of RXFP3 are restricted to relaxin-3 and its analogs, we conducted a high-throughput screening campaign against 32,021 synthetic and natural product-derived compounds using a cyclic adenosine monophosphate (cAMP) measurement-based method. Only one compound, WNN0109-C011, was identified following primary screening, secondary screening and dose-response studies. Although displayed agonistic effect in cells overexpressing the human RXFP3, it also showed cross-reactivity with the human RXFP4. This hit compound may provide not only a chemical probe to investigate the function of RXFP3/4, but also a novel scaffold for the development of RXFP3/4 agonists.

## 1. Introduction

Relaxin/insulin-like family peptide receptor 3 (RXFP3), also named as G protein-coupled receptor 135 (GPCR135) or somatostatin- and angiotensin-like peptide receptor (SALPR), is classified as a class A G protein-coupled receptor (GPCR) [1,2,3]. It is predominantly distributed in various regions of the brain, particular in the paraventricular nucleus, with low expression in peripheral tissues [1,4,5]. It holds high sequence conservation between the human and other vertebrate genomes [6,7,8,9,10,11]. Meanwhile, RXFP3 also shares 43% amino acid sequence identity and 60% similarity with its sister receptor RXFP4 [5]. Relaxin-3, a neuropeptide of the relaxin/insulin-like superfamily with two chains and three disulfide bonds, is the endogenous ligand for RXFP3 [4]. It is highly conserved across different species [11] and mainly expressed in mammalian brains [4,6,7,8,9,10,11,12,13,14,15]. RXFP3 stimulation leads to different downstream effects including inhibition of cyclic adenosine monophosphate (cAMP) production [4], elevation in phosphorylation of extracellular regulated protein kinases 1/2 (ERK1/2) [16,17,18], and promotion of β-arrestin recruitment [5,17,18].

To date, many efforts have been made to understand the physiological role of the relaxin-3/RXFP3 system. Knockout mice lacking RXFP3 display hypoactivity and a decrease in anxiety-like behavior [19], a phenomenon similar to the relaxin-3 null mutation mice [20,21]. Chronic RXFP3 activation promotes behavioral arousal in mice [22] and the food intake and body weight were enhanced by treatment with relaxin-3 or other RXFP3 agonists in rats [23,24,25,26,27,28,29,30,31], an effect that could be reversed by RXFP3 antagonists [30,31]. Moreover, RXFP3 is also postulated to modulate memory, stress, anxiety and depression [32,33,34,35], implying its potential to serve as a target for treatment of various central nervous system (CNS) diseases.

Although RXFP3 is attractive for drug discovery, most available ligands for this receptor are restricted to peptidic analogs of relaxin-3 [4,17,29,30,31,36,37,38,39]. To date, only a limited number of small molecule modulators have been identified, including a series of small molecule RXFP3/4 dual agonists [28] and a selective positive allosteric modulator [40]. Since peptidic ligands for RXFP3/4 are hard to make and no selective RXFP3 agonist has been found, we conducted a high-throughput screening (HTS) campaign against 32,021 synthetic and natural product-derived compounds using a cAMP measurement-based method. Only one compound (WNN0109-C011) that cross-reacted with RXFP4 was identified following primary screening, secondary screening and dose-response studies.

## 2. Results

### 2.1. Assay Validation

**Figure 1:**
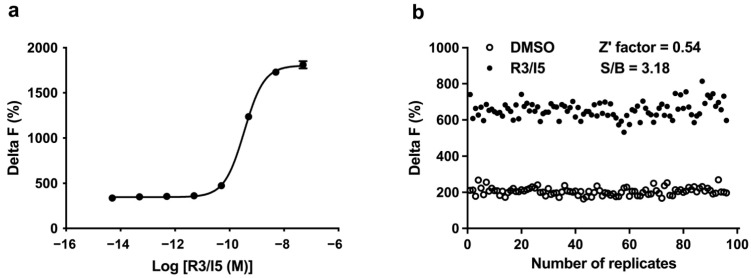
Validation of the HTS assay. (**a**) Concentration-dependent agonistic activity of R3/I5 in hRXFP3-CHO cells under the optimized assay conditions. (**b**) Z’ factor determination. A total of 192 replicates of R3/I5 and background signals were studied.

As RXFP3 is coupled to G_i_ protein to trigger the inhibition of cAMP accumulation, we applied forskolin to produce cAMP signals for the detection of inhibitory effects of small molecule compounds. The optimal concentration of forskolin was selected from dose-response curves generated at different cell densities that were also optimized during assay development. Finally, 2000 cells/well and 25 μM forskolin were used for the HTS campaign. Under the optimized conditions, the *p*EC_50_ value of R3/I5 (a RXFP3 agonist peptide) was 9.46 ± 0.02 (Figure 1a), consistent with that documented in the literature [37].

### 2.2. Assay Performance

In the HTS campaign with cAMP accumulation assay, the positive signals were evoked by R3/I5, a chimeric peptidic agonist for RXFP3, while the negative signals (background) were assessed in the presence of 1% DMSO. As shown in Figure 1b, coefficient of variation (CV) values were 7.18% (for R3/I5) and 10.37% (for background), respectively. Z’ factor calculated was 0.54 with an S/B ratio of 3.18. These parameters are indicative of a high assay quality that is suitable for HTS [41].

### 2.3. Lead Identification

**Figure 2:**
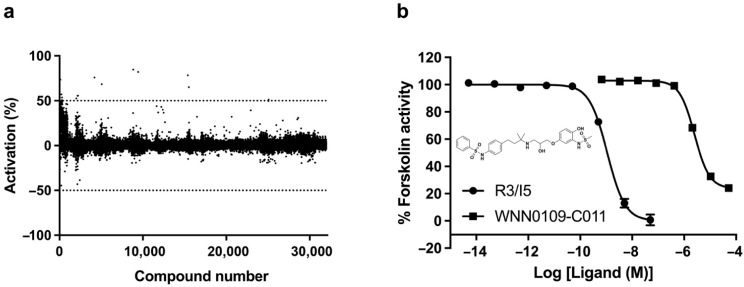
HTS campaign to discover potential RXFP3 agonists. (**a**) Large-scale screening of 32,021 synthetic and natural product-derived compounds. (**b**) Chemical structure of WNN0109-C011 and activation of human RXFP3 by WNN0109-C011.

**Figure 3:**
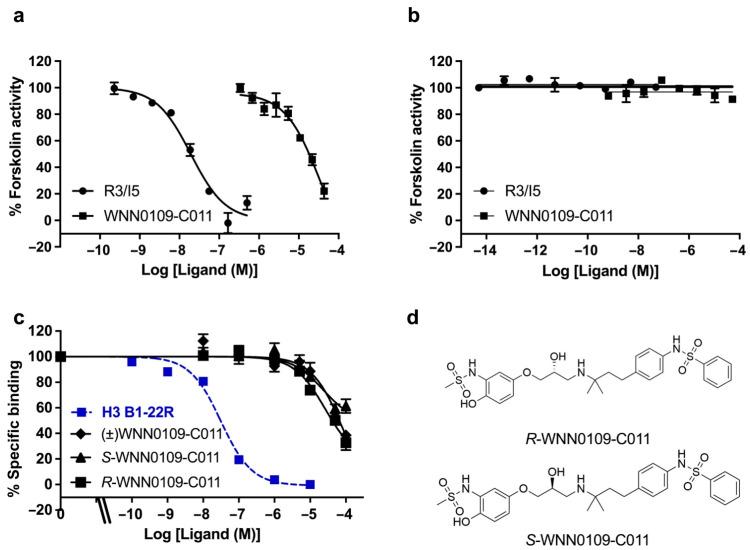
Specificity of WNN0109-C011. (**a**) WNN0109-C011 displayed an agonistic effect on hRXFP3-CHO cells. (**b**) WNN0109-C011 did not activate parental CHO cells. (**c**) Competitive binding of europium-labelled H3 B1-22R with WNN0109-C011 and its two enantiomers (*S*-WNN0109-C011 and *R*-WNN0109-C011) in hRXFP3-CHO cells. (**d**) Chemical structures of the two enantiomers of WNN0109-C011. Data shown are means ± SEM.

As displayed in Figure 2a and Supplementary Table S1, nineteen hits were identified initially during primary screening with more than 50% activation. Among them, 4 scaffolds were identified, including quinoxaline-2,3-dione derivatives (WNN0002-F006 and WNN0119-H005), 1,4-dihydrothieno [2,3-b] pyrazine-2,3-dione derivatives (WNN0003-G011, WNN0004-D007, WNN0003-F007 and WNN0003-B006), tricyclic derivatives (WNN0314-G003, WNN0195-H002 and WNN0314-F004), and 10,11-dihydro-5H-dibenzo [a, d] [7] annulene derivatives (WNN0063-E009 and WNN0063-E008), with activation profiles ranging between 50% and 130% compared with R3/I5 (positive control). Of which, WNN0063-E009 exhibited the best agonistic effect as opposed its 3-carboxylic acid piperidine analog WNN0063-E008. Subsequent screening and dose-response studies confirmed only one hit (WNN0109-C011; Figure 2b) with no structural resemblance to the hits identified in the HTS campaign against human RXFP4 [42]. Obviously, the structure of WNN0109-C011 is not similar to that of the selective RXFP4 agonist compound 14b, indicating that they may interact with different sites of RXFP4. Indeed, WNN0109-C011 displayed *p*EC_50_ values of 5.60 ± 0.02 for human RXFP3 (Figure 2b) and 4.47 ± 0.21 for human RXFP4 (Figure 3a) in the cAMP accumulation assay. It did not activate parental CHO cells (Figure 3b), suggesting that it is a nonselective RXFP3/4 agonist. The europium-labelled H3 B1-22R competition binding assay further confirmed the direct binding of WNN0109-C011 and its two enantiomers with RXFP3 (Figure 3c,d, Supplementary Table S2).

### 2.4. Binding Pose Prediction

**Figure 4:**
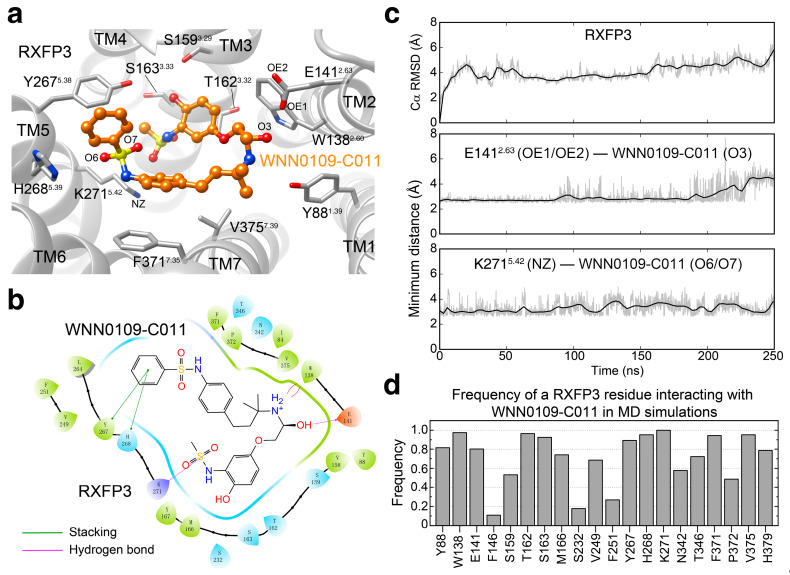
Predicted binding pose of WNN0109-C011 in RXFP3 by molecular docking and molecular dynamics (MD) simulation studies. (**a**) Final snapshot of the binding pose of WNN0109-C011 in RXFP3 after 250 ns MD simulations. (**b**) The WNN0109-C011−RXFP3 interaction diagram. WNN0109-C011 is shown as stick. Residues are represented as spheres and colored by interaction type. Interactions between residue and ligand atoms are drawn as lines, colored by interaction type. (**c**) Analysis of the MD simulations of RXFP3 bound by WNN0109-C011: top, root mean square deviation (RMSD) of Cα positions of RXFP3, where all snapshots were superimposed on the homology model of active RXFP3 (downloaded from GPCRdb website) using the Cα atoms; middle, minimum distance between O3 atom of WNN0109-C011 and the negatively charged atoms of E141 ^2.63^; bottom, minimum distance between O6/O7 atoms of WNN0109-C011 and the positively charged atom of K271 ^5.42^. (**d**) Frequency of a RXFP3 residue interacting with WNN0109-C011 during the last 150 ns MD simulations. The frequency value indicates the stability of a particular residue−ligand interaction. The interaction is defined by the heavy atom distance between the residue and ligand using 4.0 Å as cutoff.

To reveal the potential binding mode of WNN0109-C011 in RXFP3, we performed molecular docking and molecular dynamics (MD) simulation studies. As shown in Figure 4, WNN0109-C011 stably occupied the orthosteric binding pocket formed by transmembrane helices (TMs) 2, 3, 5, 6, and 7. Specifically, the terminal group methanesulfonamide inserted deeply to the ligand-binding pocket with the formation of a hydrogen bond with K271 ^5.42^ (superscript refers to Ballesteros–Weinstein numbering [43]), as seen from the measured minimum distance between the specific atoms of K271 and WNN0109-C011 (Figure 4c). The middle hydroxy pointed toward the side chains of E141 ^2.63^ with the formation of one hydrogen bond. Meanwhile, the adjacent charged amide was stabilized by the stacking interaction with W138 ^2.60^. Another terminal group benzene located closely to TM5 with the formation of rich stacking interactions with Y267 ^5.38^ and H268 ^5.39^. Besides the above interactions, massive hydrophobic contacts contributed by M166 ^3.36^, V249 ^ECL2^, F251 ^ECL2^, F371 ^7.35^, P372 ^7.36^ and V375 ^7.39^ were observed. Among the twenty RXFP3 ligand-binding pocket residues (Figure 4d), RXFP4 utilizes identical or similar amino acids at 12 positions (Y46 ^1.39^, W97 ^2.60^, E100 ^2.63^, F105 ^ECL1^, T121 ^3.32^, F195 ^ECL2^, Y204 ^5.38^, N262 ^6.51^, T266 ^6.55^, F291 ^7.35^, P292 ^7.36^ and H299 ^7.43^) and 4 positions (V125 ^3.36^, T176 ^4.60^, L193 ^ECL2^ and R208 ^5.42^), respectively. Of note, four residues that contribute strong interactions with WNN0109-C011 are different between RXFP4 and RXFP3 (L118 ^3.29^, V122 ^3.33^, Q205 ^5.39^ and T295 ^7.39^ for RXFP4, and S159 ^3.29^, S163 ^3.33^, H268 ^5.39^ and V375 ^7.39^ for RXFP3), which may explain the reduced potency of WNN0109-C011 at RXFP4.

## 3. Discussion

Previous studies have shown that the cognate ligand of RXFP3, relaxin-3, plays regulatory roles in appetite, arousal, memory, stress, anxiety and depression [19,20,21,22,23,24,25,26,27,28,29,30,31,32,33,34,35], thereby making this receptor an attractive target for drug discovery. Given the structural complexities of relaxin-3 and its peptide analogs and the technical challenges in their syntheses together with their lack of blood brain barrier penetration, small molecule agonists for RXFP3 are thus in high demand. Up to now, there exist only a limited number of small molecule RXFP3 modulators [28,40], among which, none demonstrate RXFP3 specificity.

In this study, we established an HTS assay by detecting cAMP accumulation in CHO cells stably expressing RXFP3, a method that was used to screen a collection of 32,021 synthetic and natural product-derived compounds against RXFP3. The RXFP3 agonist peptide R3/I5 was used as a positive control to optimize the assay system, and our results (Figure 1) showed that the assay parameters, such as CV, S/B ratio and Z’ factor, were all of high quality. Although the HTS campaign identified 23 initial hits (0.07%) most of them failed to show agonistic activities in secondary screening or dose response studies, leading to only one confirmed hit (WNN0109-C011). This compound, with no structural resemblance to the hits identified in the HTS campaign against human (RXFP4) [42], was subsequently found to inhibit forskolin-elicited cAMP responses in both hRXFP3-CHO and hRXFP4-CHO cells, without affecting cAMP levels in parental CHO cells, demonstrating its dual agonist (RXFP3/4) potential.

## 4. Materials and Methods

### 4.1. Materials

R3/I5 was obtained from Phoenix Pharmaceuticals (Burlingame, CA, USA). cAMP dynamic kit was bought from Cisbio Bioassays (Codolet, France) and white 384-well plates were from PerkinElmer (Waltham, MA, USA). Forskolin, 3-isobutyl-1-methylxanthine (IBMX), dimethyl sulfoxide (DMSO) and bovine serum albumin (BSA) were purchased from Sigma-Aldrich (St. Louis, MO, USA). Dulbecco’s modified Eagle’s medium/nutrient mixture F-12 (DMEM/F12), Hank’s balanced salt solution (HBSS), L-glutamine, penicillin-streptomycin, 0.25% trypsin-EDTA, Dulbecco’s phosphate-buffered saline (DPBS) and fetal bovine serum (FBS) were all supplied by Life Technologies (Carlsbad, CA, USA).

### 4.2. Cell Culture

Chinese hamster ovary (CHO) cells stably expressing the human RXFP3 (hRXFP3-CHO) [44] or RXFP4 (hRXFP4-CHO) [45] and their parental CHO cells were maintained in DMEM/F12 supplemented with 10% (*v/v*) FBS, 2 mM L-glutamine, 100 units/mL penicillin and 100 μg/mL streptomycin at 37 °C in 5% CO_2_.

### 4.3. Compound Library

A collection of 32,021 synthetic and natural product-derived compounds stored at the Chinese National Compound Library [46] were used in this study. The structural diversity covers heterocycles, lactams, sulfonates, sulfonamides, amines and secondary amides, etc. All the compounds are highly pure, and the stock, pre-solubilized in 100% DMSO solution, was applied to the primary screening. 

### 4.4. HTS Campaign

The HTS campaign was carried out in white 384-well plates by measuring the inhibition of forskolin-stimulated cAMP accumulation in hRXFP3-CHO cells. Briefly, the positive control (5 nM R3/I5) and negative control (1% DMSO) were located at the 64 wells of the outer four columns on both sides with 32 replicates, respectively. The individual screening compounds were loaded at the center twenty columns with an average final concentration of 10 μM in a single-dose format without replicates. Time-resolved fluorescence resonance energy transfer (TR-FRET) signals were read by an EnVision multilabel plate reader (PerkinElmer). Z’ factor, coefficient of variation (CV) and signal-to-background ratio (S/B) were calculated according to the literature [41]. Positive control was regarded as 100% activation, while negative control represented no activation. Compounds showing greater than 50% activation were considered hits, and secondary screening using the same assay was performed to eliminate false positives.

### 4.5. cAMP Assay

cAMP accumulation assay was performed in hRXFP3-CHO cells at a density of 5 × 10^5^ cells/mL. The cells were treated with different concentrations of compounds plus 25 μM forskolin and 500 μM IBMX for 30 min at room temperature (RT). cAMP-d2 conjugate and cryptate conjugate working solutions were then diluted and added to the plates separately. Plates were incubated at RT for 60 min and the fluorescence intensity measured at 620 nm and 665 nm by an EnVision multilabel plate reader. The parental CHO cells were applied to examine off-target effect, while hRXFP4-CHO cells were utilized for selectivity assessment.

### 4.6. Europium-Labelled H3 B1-22R Competition Binding

CHO-K1 cells stably transfected with RXFP3 were plated onto pre-coated poly-L-lysine 96-well plates for the binding assay performed using 5 nM europium (Eu)-H3 B1-22R as previously described [46] with triplicate determinations within assay and a minimum of three independent experiments. Data are presented as means ± SEM. *pKi* values were calculated using one-site fit *Ki* and a *Kd* value of 26 nM for H3 B1-22R [47].

### 4.7. Molecular Docking

Molecular docking was performed with Schrödinger Suite 2018−1 (New York, NY, USA). Homology model of active hRXFP3 retrieved from the GPCRdb website [48] was built mainly based on the X-ray structure of the active angiotensin II type 1 receptor (PDB code: 6DO1) with a sequence similarity of 34%. Receptor structure and ligands were prepared by Protein Preparation Wizard and LigPrep in Schrödinger Suite 2018−1 with default settings, respectively. Receptor grid was generated by Receptor Grid Generation tool in the Glide (New York, NY, USA) [49]. Ligands were docked following the XP scoring function. The initial docking poses were visualized and analyzed, while ten poses with best docking scoring scores were subjected to Prime MM-GBSA optimization by Prime MM-GBSA module (New York, NY, USA). Finally, by visual inspection of the optimized docking pose and considering the XP docking scores, two predicted binding poses of WNN0109-C011 in hRXFP3 were obtained for MD simulations.

### 4.8. Molecular Dynamics Simulation

MD simulation study was performed by Gromacs 2020.1 (Groningen, The Netherlands) [50]. The hRXFP3 structure was capped using CHARMM-GUI Membrane Builder v3.2.2 (Bethlehem, PA, USA) [51]. Residue D128 ^2.50^ was deprotonated, while other titratable residues were left in their dominant state at pH 7.0. The WNN0109-C011−RXFP3 complexes were embedded in a bilayer composed of 201 POPC lipids and solvated with 0.15 M NaCl in explicit TIP3P waters using CHARMM-GUI Membrane Builder v3.2.2 (Bethlehem, PA, USA) [51]. The CHARMM36-CAMP force filed [52] was adopted for protein, lipids and salt ions. WNN0109-C011 was modelled with the CHARMM CGenFF small-molecule force field [53]. The Particle Mesh Ewald (PME) method was used to treat all electrostatic interactions beyond a cut-off of 12 Å and the bonds involving hydrogen atoms were constrained using LINCS algorithm [54]. The complex system was first relaxed using the steepest descent energy minimization, followed by slow heating of the system to 310 K with restraints. The restraints were reduced gradually over 20 ns. Finally, 250 ns restrain-free production run was carried out, with a time step of 2 fs in the NPT ensemble at 310 K and 1 bar using the Nose-Hoover thermostat and the semi-isotropic Parrinello-Rahman barostat [55], respectively.

### 4.9. Statistical Analysis

Dose-response data were analyzed with Prism software (GraphPad, San Diego, CA, USA) using a sigmoidal model with variable slope. Data were expressed as the means ± S.E.M of at least three independent experiments.

## 5. Conclusions

In summary, we have developed an optimized HTS method by measuring cAMP production to identify novel RXFP3 agonists. Our HTS campaign and follow-up hit characterization experiments identified WNN0109-C011 as a potential agonist for both RXFP3 and RXFP4, without any agonistic effect on parental CHO cells. Discovery of specific RXFP3 agonists is still challenging along with an urgency of in-depth evaluation of its in vivo functions associated with the druggability of this target. Determination of 3-dimensiaonl structures of both RXFP3 and RXFP4 would certainly significantly advance this important research field.

## Data Availability

Data is contained within the article or supplementary material.

## Supplementary Materials

The following are available, Table S1: Hits from primary screening; Table S2: Binding of europium-labelled H3 B1-22R to RXFP3 in competition with various small molecules agonists.
