# Supplementary material for: High-Throughput Screening Campaign Identified a Potential Small Molecule RXFP3/4 Agonist
Source: Molecules. 2021 Dec 11;26(24):7511. doi: 10.3390/molecules26247511 (PMC8709172; doi:10.3390/molecules26247511)
Supplement: Supplementary file 1 [file molecules-26-07511-s001.zip › molecules-1447788-supplementary.pdf]

Supplementary Table S1. Hits from primary screening.

Compound	Structure	Activity (% positive control)
WNN0002-F006	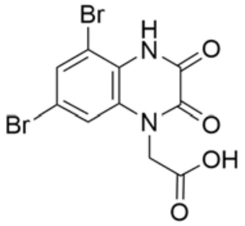	73
WNN0003-B006	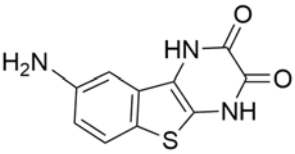	54
WNN0003-F007	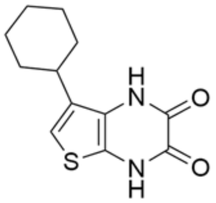	51
WNN0003-G011	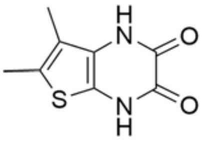	54
WNN0004-D007	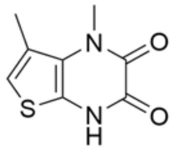	57
WNN0027-E003	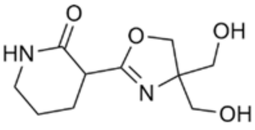	53
WNN0027-G008	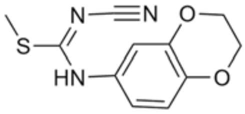	56
WNN0028-D009	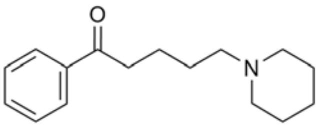	56
WNN0048-A002	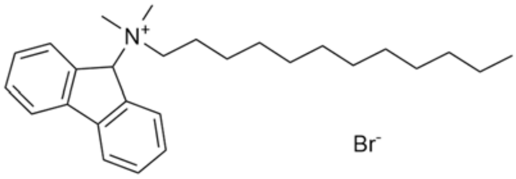	215

WNN0056-B006	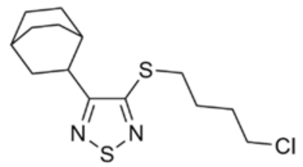	76
WNN0063-E008	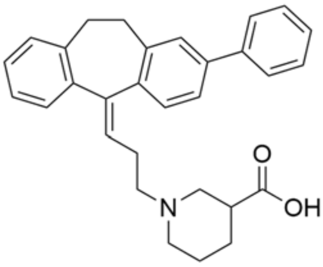	68
WNN0063-E009	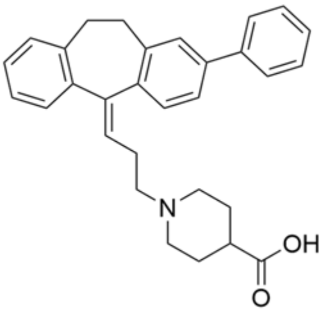	130
WNN0109-C011	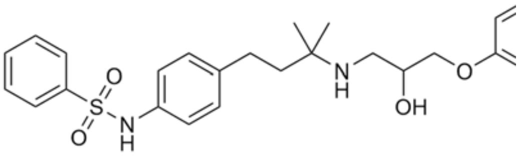	85
WNN0119-H005	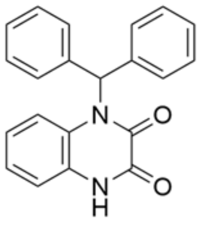	82
WNN0148-E005	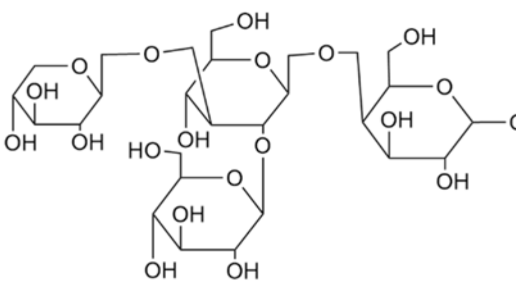	129
WNN0195-H002	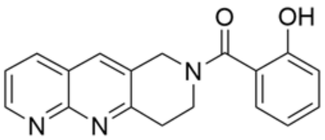	65

WNN0196-B004	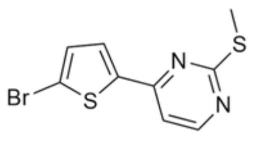	78
WNN0314-F004	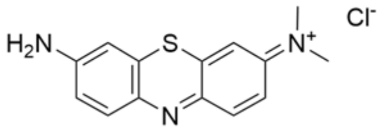	50
WNN0314-G003	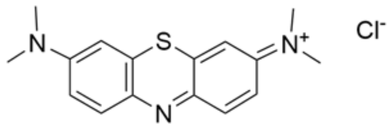	51

Supplementary Table S2. Binding of europium-labelled H3 B1-22R to RXFP3 in competition with various small molecules agonists.

Compound	*p*Ki
H3 B1-22R (RXFP3 antagonist)	7.59 ± 0.06 (n=12)
R-WNN0109-C011	4.50 ± 0.16 (n=3)
S-WNN0109-C011	4.04 ± 0.16 (n=3)
(±)WNN0109-C011	4.38 ± 0.10 (n=3)
